# Injuries of the Eye, Orbit, and Eyelids; Their Immediate and Remote Effects

**Published:** 1867-10

**Authors:** 


Injuries of the Eye, Orbit, and Eyelids; Their Immediate and Remote Effects. By George Lawson, F.R.C.S., Eng., Assistant-Surgeon to the Royal London Ophthalmic Hospital, Moorfields; and to the Middlesex Hospital; late Assistant-Surgeon, Rifle Brigade. With numerous Illustrations. Philadelphia: Henry C. Lea. 1867.

This is a moderate-sized octavo volume, of 408 pages, neatly published and well illustrated with cuts. It is an American edition of a new English work, of a strictly surgical character, and on topics of much practical importance. The contents embrace the consideration of Superficial Injuries of the Eye; Injuries of the Eye from Burns, Scalds, and Chemical Agents; Penetrating Wounds of the Eye, and other Injuries of the Cornea and Iris; Traumatic Cataract; Capsular Opacities and Dislocations of the Lens; Foreign Bodies within the Eye; Traumatic Intraocular Hemorrhage and Rupture of the Globe; Gunshot Injuries of the Eye; Sympathetic Ophthalmia; Injuries of the Orbit; Injuries of the Eyelids; Abstract of the Surgical Report of the Ophthalmic Hospital, Moorfields; and Jæger's Test Types. The reader will thus see the general scope of the work. We have not yet had time to examine it sufficiently to judge of its special merits, either as to style or matter.

For sale by S. C. Griggs & Co., 39 and 41 Lake Street, Chicago.

